# Clinicopathological and prognostic values of fibronectin and integrin αvβ3 expression in primary osteosarcoma

**DOI:** 10.1186/s12957-019-1566-z

**Published:** 2019-01-28

**Authors:** Kai Shi, Sheng-lin Wang, Bin Shen, Feng-qiang Yu, Dan-feng Weng, Jian-hua Lin

**Affiliations:** 1 Department of Orthopaedics, the First Affiliated Hospital of Fujian Medical University, Fuzhou, 350005 Fujian People’s Republic of China; 2 Faculty of Education, the Chinese University of Hong Kong, Shatin, N.T., 999077 Hong Kong People’s Republic of China; 3 Department of Pathology, the First Affiliated Hospital of Fujian Medical University, Fuzhou, 350005 Fujian People’s Republic of China; 4 Department of Central Laboratory, the First Affiliated Hospital of Fujian Medical University, Fuzhou, 350005 Fujian People’s Republic of China

**Keywords:** Osteosarcoma, Fibronectin, αvβ3, Co-expression, Prognosis

## Abstract

**Background:**

Osteosarcoma is a malignant bone tumor with a high potential for lung metastasis, and the prognosis for patients with metastatic disease is very poor. The interaction between fibronectin (FN) and integrin αvβ3 in soft-tissue sarcoma promotes cell migration, invasion, and lung metastasis. This study aimed to investigate the prognostic significance of FN and αvβ3 in osteosarcoma.

**Methods:**

Immunohistochemistry and western blotting were used to detect the expression of FN and αvβ3 in 60 osteosarcoma specimens and in 30 osteochondroma specimens. Furthermore, correlations of FN and αvβ3 with the clinicopathological features of osteosarcoma patients were analyzed using the *χ*^2^ test and Fisher’s exact test. Disease-free survival and overall survival of osteosarcoma patients were assessed using the Kaplan-Meier method and Cox proportional hazards model. The predictive accuracy of the model was determined by the Harrell concordance index.

**Results:**

FN (*P* < 0.05) and αvβ3 (*P* < 0.05) were overexpressed in osteosarcoma specimens compared with osteochondroma specimens. High FN expression was associated with a poor response to chemotherapy (*P* = 0.001) and poor disease-free (*P* < 0.001) and overall (*P* < 0.001) survival. High expression of αvβ3 was linked to an advanced surgical stage (*P* = 0.028), a poor response to chemotherapy (*P* = 0.002), and both poor disease-free survival (*P* < 0.001) and overall survival (*P* < 0.001). FN and αvβ3 co-expression were associated with sex (*P* = 0.011), an advanced surgical stage (*P* = 0.013), and a poor response to chemotherapy (*P* = 0.002). Moreover, high expression of both proteins can serve as an independent prognostic value for reduced survival time in osteosarcoma patients.

**Conclusions:**

The results of this study suggest that FN and αvβ3 expression is associated with an unfavorable clinical outcome of osteosarcoma, and these molecules may constitute attractive therapeutic targets for osteosarcoma treatment. To improve the survival of osteosarcoma patients, further investigations are required to clarify their prognostic values in a larger population.

**Electronic supplementary material:**

The online version of this article (10.1186/s12957-019-1566-z) contains supplementary material, which is available to authorized users.

## Introduction

As the most frequently observed primary aggressive bone tumor, osteosarcoma occurs most often in childhood and adolescence, with a second incidence peak among individuals over 50 years of age [1]. Aggressive therapeutic modalities, including surgical resection and combinational chemotherapy, can cure 70% of patients with localized disease. However, the prognosis of patients with metastatic or relapsed osteosarcoma remains unfavorable, with no improvement over the past 30 years [1]. Thus, further investigation of the biomarkers for the prognosis of osteosarcoma is needed to develop effective agents for treatment.

In cancer development and progression, the extracellular matrix (ECM) undergoes compositional and organizational remodeling and facilitates tumor angiogenesis by regulating the dynamic behaviors of endothelial cells through various cell adhesion receptors. Accordingly, ECM proteins are potentially promising therapeutic targets [2].

Fibronectin (FN) is a multifunctional glycoprotein of the ECM that plays a crucial role in cell adhesion and angiogenesis. In the process of metastatic progression, FN acts as a potent guidance and motility cure for cancer cells via ECM remodeling and ECM-guided directional migration [3]; FN independently indicates unfavorable clinical outcomes in nasopharyngeal carcinoma [4] and head and neck squamous cell carcinomas [5]. As a bone matrix protein synthesized by osteoblasts, FN also regulates the differentiation and survival of osteoblasts [6]. Overall, high FN levels of expression are observed in osteosarcoma cell lines [7].

Integrins are cell adhesion receptors mediating tumor cell migration, proliferation, and invasion through recognition of diverse matrix ligands, including FN, collagen, and laminin [8]. Among members of the integrin family, integrin αvβ3 specifically binds to FN with high affinity [9]. Integrin αvβ3 expression is strongly increased in tumor cells, with a prominent role as a pro-angiogenic factor in the progression of various tumor types [10]. Upregulation of αvβ3 integrin is also involved in the exogenously induced cell migration, invasion, and anti-apoptotic activity of osteosarcoma cells [11]. Targeted imaging of integrin αvβ3 can be employed to specifically detect tumor location and size in osteosarcoma and may provide a potential tool in pre-operative assistance or therapy monitoring [12].

Interaction between FN and αvβ3 contributes to osteoblast adhesion and proliferation [13]. Furthermore, depletion of αvβ3 in osteosarcoma cells reduces cell adhesion and spread on FN [14]. However, the prognostic impact of FN and αvβ3 on osteosarcoma has yet to be explored. In this study, we analyzed FN and αvβ3 expression levels via immunohistochemistry and western blotting and examined correlation of the individual expression as well as co-expression with the clinicopathological features, disease-free survival (DFS), and overall survival (OS) of patients with osteosarcoma to identify the potential clinicopathological and prognostic values of these factors in osteosarcoma.

## Materials and methods

### Patients and tissue specimens

We carried out a retrospective study of 60 patients with primary osteosarcoma who had undergone complete surgical resection at the First Affiliated Hospital of Fujian Medical University between 2009 and 2014. Histopathological diagnosis of all specimens was confirmed by a senior doctor of pathology. All patients underwent standardized neoadjuvant and postoperative chemotherapy with ifosfamide, cisplatin, and doxorubicin. Relevant clinical data were retrieved from medical records, including sex, age at diagnosis, tumor size, tumor location, histologic subtype, Enneking staging, and response to chemotherapy. Formalin-fixed and paraffin-embedded surgical tumor specimens for immunohistochemical staining were obtained from the archives of the Department of Pathology.

Follow-up of osteosarcoma patients was terminated on 31 August 2017 either by phone call or outpatient visit. To assess the development of local recurrence and distant metastasis, all patients with osteosarcoma were monitored by X-ray or lung computed tomography (CT) scans after surgical excision every 3 months during the first 3 years and every 6 months thereafter. DFS time was calculated from the date of diagnosis until the date of first tumor progression. OS time was calculated from the date of diagnosis until the date of death. Patients were censored at the date of the last follow-up if tumor progression or death had not occurred.

Written informed consent was provided by all participants involved in this study. The study protocol conformed to the ethical guidelines of the Declaration of Helsinki and was approved by the Ethics Committee of the First Affiliated Hospital of Fujian Medical University.

### Immunohistochemistry and scoring

Immunohistochemical expression of FN and αvβ3 in archival osteosarcoma specimens were examined by applying a PV-9000 Polymer Detection System (Zhongshan Goldenbridge Inc., Beijing, China), with 30 corresponding osteochondroma tissues which were resected at the First Affiliated Hospital of Fujian Medical University between 2009 and 2014 used as controls. Paraffin-embedded specimens were serially sectioned (4 μm) and incubated for 1 h at 60 °C. The tissue sections were then deparaffinized, hydrated, and incubated with 3% hydrogen peroxide for 10 min at room temperature to block endogenous peroxidase activity. During the antigen retrieval process, the sections were placed in citrate buffer (pH 6.0) in an electromagnetic oven for 2 min and then allowed to cool to room temperature. The sections were incubated overnight at 4 °C with antibodies against FN (mouse monoclonal 2755-8; 1:50; Santa Cruz, USA) and αvβ3 (rabbit polyclonal orb10927; 1:50; Biorbyt, UK). Next, the sections were incubated with a polymer helper reagent for 20 min at room temperature and then poly-peroxidase-anti-mouse/rabbit IgG for 30 min at room temperature according to the manufacturer’s instructions. After staining with diaminobenzidine (Zhongshan Goldenbridge Inc.), the sections were counterstained with hematoxylin, dehydrated, and mounted. Negative (PBS (0.01 M, pH 7.2) rather than primary antibodies) and known positive (human esophagus tissue for FN and human lung cancer tissue for αvβ3) controls were stained in parallel with each set of sections studied.

The staining results were evaluated by two independent observers (CYP and ZZZ). Cytoplasmic staining for FN or αvβ3 in tumor cells was interpreted as a positive result. The average labelling index of FN from five random high-power fields was semi-quantitatively recorded as follows: 0, no positive staining; ±, only a few scattered positive cells accounting for less than 20% of tumor cells; +, cluster(s) of positively stained cells accounting for 20–30% of tumor cells; ++, cluster(s) of positively stained cells accounting for greater than 30% of tumor cells [15]. The average labelling index of αvβ3 from five random high-power fields was recorded as follows: 0, absent; ±, weak expression, accounting for greater than 20% of tumor cells; +, moderate expression, accounting for greater than 20% of tumor cells; ++, strong expression, cells accounting for greater than 20% of tumor cells [16]. Specimens showing immunostaining of ++ were defined as high expression of FN or αvβ3; expression levels of ± or + were defined as low expression, and 0 as negative expression.

### Western blotting

Tissues (100 mg) of 60 osteosarcoma and 30 corresponding osteochondroma cases were ground into powder in liquid nitrogen and lysed in lysis buffer (cat. no. G2002; Servicebio Technology, Wuhan, China). Protein concentrations in the lysates were then quantitated using a Bicinchoninic Acid Protein Assay kit (cat. no. G2026; Servicebio Technology) and preserved at − 80 °C. Proteins (40 μg) were separated by 10% SDS-PAGE and transferred to PVDF membranes. The membranes were incubated with primary anti-FN (1:1000; Santa Cruz) and anti-αvβ3 (1:1000; Biorbyt) antibodies overnight at 4 °C, followed by incubation with a horseradish peroxidase-conjugated secondary antibody (cat. no. GB23404; 1:3000; Servicebio Technology) for 1 h at 37 °C. The signal was visualized using an Enhanced Chemiluminescence detection system (Amersham Biosciences, UK) and Image Lab software version 3.0 (Bio-Rad Laboratories, Inc., USA). β-actin was simultaneously detected using mouse anti-β-actin antibody (1:5000; Servicebio Technology) as a loading control.

### Statistical analysis

The chi-square test, Fisher’s exact test, or Student’s *t t*es*t* (independent-sample) was used to compare FN and αvβ3 expression between osteosarcoma and osteochondroma and to determine whether their expression was correlated with the clinicopathological data of the osteosarcoma patients. Spearman’s rank coefficient was applied to determine the correlation between FN and αvβ3 expression. Kaplan-Meier survival plots were employed for univariate analysis and the log-rank test was utilized to compare differences in survival distributions. The Cox proportional hazards model was used to perform multivariate analysis for all parameters significant in the univariate analysis. The Harrell concordance index (C-index) was calculated to measure the performance of the model. All statistical analyses were performed using SPSS software version 19.0 (SPSS Inc., Chicago, USA). *P* < 0.05 was considered statistically significant.

## Results

### Expression of FN and αvβ3 in osteosarcoma and osteochondroma specimens

**Fig. 1:**
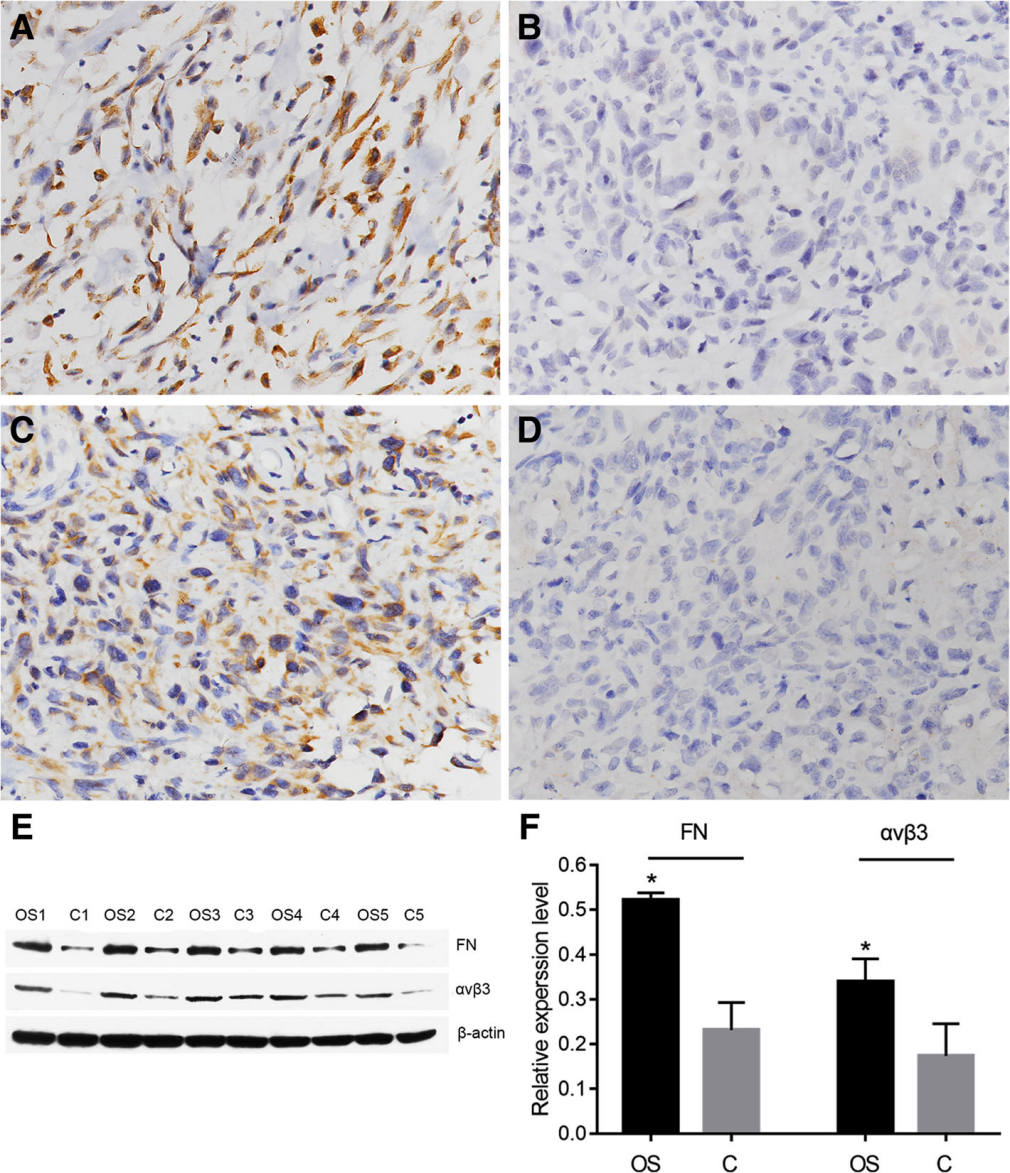
Expression of FN and αvβ3 in osteosarcoma and osteochondroma specimens. Representative images of immunohistochemistry show high cytoplasmic FN expression in osteosarcoma (**a**) and low FN expression in osteochondroma (**b**) as well as high cytoplasmic αvβ3 expression in osteosarcoma (**c**) and low αvβ3 expression in osteochondroma (**d**). Original magnification, × 200. **e** Representative images of western blotting show the expressions of FN and αvβ3 in the lysed osteosarcoma and osteochondroma. **f** Quantification of expression levels of FN and αvβ3 in osteosarcoma and osteochondroma tissues. β-actin was used as an internal loading control. OS, osteosarcoma; C, osteochondroma. Columns, mean from 60 or 30 tissues; bars, square deviation (*P < 0.05 by an independent-sample *t* test)

**Fig. 2:**
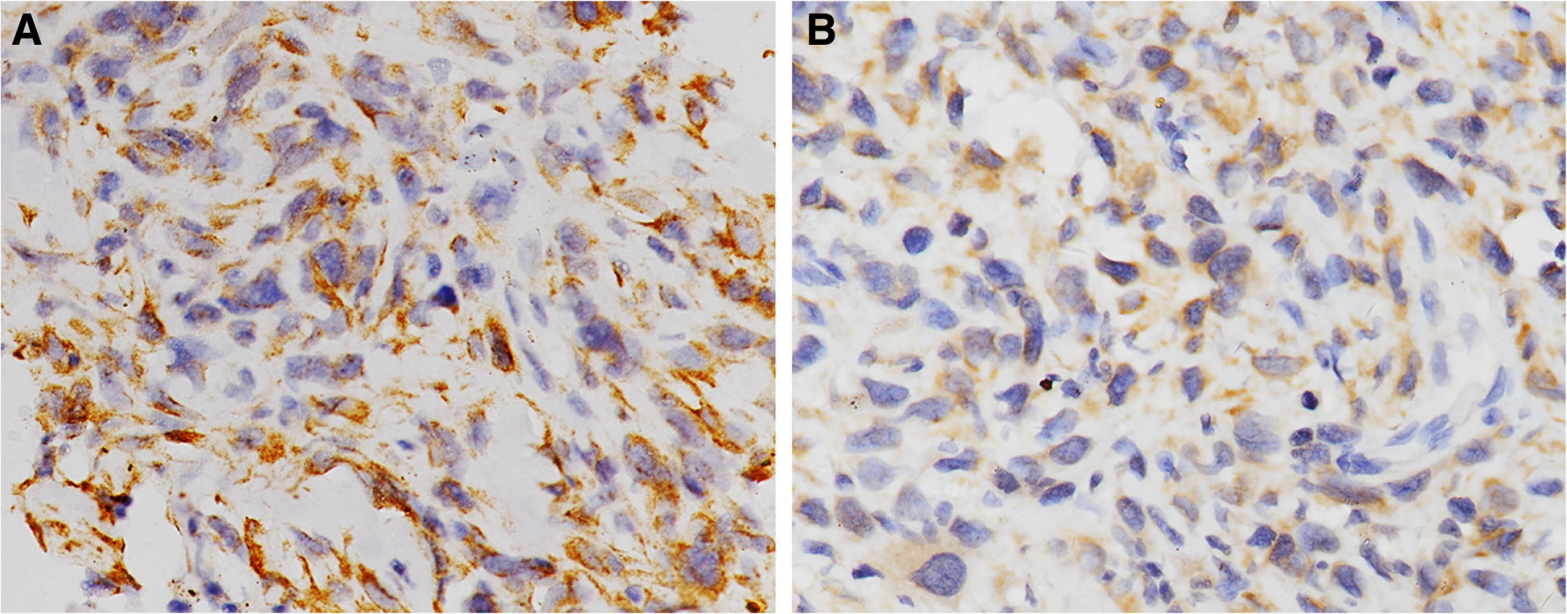
Co-expression of FN and αvβ3 in one osteosarcoma specimen. Immunohistochemical staining showed high expression of FN (**a**) and αvβ3 (**b**) in one osteosarcoma specimen. Original magnification, × 200

FN and αvβ3 protein distribution were primarily observed in the cytoplasm of tumor cells (Fig. 1). The statistical results of immunohistochemistry are summarized in Table 1. FN and integrin αvβ3 were highly expressed in 19 (31.7%) and 16 (26.7%) of 60 osteosarcoma cases, which were not observed in the 30 osteochondroma cases. FN (*P* = 0.002) and αvβ3 (*P* < 0.001) showed higher rates of expression in osteosarcoma than in osteochondroma. The expressional levels of the two proteins were further verified by western blotting analysis. Similarly, the expression of FN (*P* < 0.001) and αvβ3 (*P* = 0.003) was also found to be upregulated in osteosarcoma tissues compared with osteochondroma tissues (Fig. 1). These results demonstrated that the expressional levels of FN and αvβ3 proteins were markedly increased in osteosarcoma tissues compared with the corresponding osteochondroma tissues. Furthermore, high expression (FN^+^/αvβ3^+^; Table 1; Fig. 2) and low/negative expression (FN^−^/αvβ3^−^; Table 1) of both FN and αvβ3 were identified in 10 and 50 osteosarcoma cases, respectively. The co-expression rates of the two proteins were significantly different in osteosarcoma and osteochondroma specimens (*P* < 0.001). A significant correlation for the level of expression was observed between FN and αvβ3 in osteosarcoma (*r* = 0.379, *P* = 0.003, Table 2).

**Table 1:** Expression of FN and αvβ3 in osteosarcoma and corresponding osteochondroma

	Osteosarcoma, *n* (%)	Osteochondroma, *n* (%)	*P* value
FN			
High expression	19 (31.7)	0 (0)	0.002
Low expression	22 (36.6)	14 (46.7)	
Negative expression	19 (31.7)	16 (53.3)	
αvβ3			
High expression	16 (26.7)	0 (0)	< 0.001
Low expression	24 (40.0)	8 (26.7)	
Negative expression	20 (33.3)	22 (73.3)	
FN^+^/αvβ3^+^	10 (16.7)	0 (0)	< 0.001
Others^a^	15 (25.0)	0 (0)	
FN^−^/αvβ3^−^	35 (58.3)	30 (100.0)	

^+^High expression; ^−^low/negative expression; ^a^FN^+^/αvβ3^−^ plus FN^−^/αvβ3^+^

**Table 2:** Correlation between FN and αvβ3 expression in osteosarcoma

FN	αvβ3			*r*	*P* value
	High expression	Low expression	Negative expression		
High expression	10	5	4	0.379	0.003
Low expression	3	14	5		
Negative expression	3	5	11		

*r* correlation coefficient

### Association between expression of FN and αvβ3 in osteosarcoma and clinicopathological characteristics

The osteosarcoma patients’ clinicopathological characteristics are summarized in the Additional file 1: Table S1. Association of FN and αvβ3 expression individually (Table 3) and together (Table 4) with clinicopathological parameters, including sex, age, tumor size, tumor location, histologic subtype, Enneking staging, and response to chemotherapy, were analyzed. Expression of FN and integrin αvβ3 was stratified according to conventional (osteoblastic, chondroblastic, and fibroblastic types) and special (small cell and telangiectatic types) osteosarcoma, rather than each histological subtype. In osteosarcoma, high FN expression was significantly associated with a poor response to chemotherapy (*P* = 0.001; Table 3), whereas high expression of αvβ3 was significantly associated with advanced Enneking staging (*P* = 0.028; Table 3) and a poor response to chemotherapy (*P* = 0.002; Table 3). Tumors were more likely to develop to an advanced surgical stage (*P* = 0.013; Table 4) and exhibit a worse response to chemotherapy (*P* = 0.002; Table 4) as the combined expression levels progressed from FN^−^/αvβ3^−^ to other groups (FN^+^/αvβ3^−^ plus FN^−^/αvβ3^+^) and then an FN^+^/αvβ3^+^ status. In addition, a sex difference (*P* = 0.011) was found among the three expression levels of FN and αvβ3.

**Table 3:** Association between individual FN and αvβ3 expression and clinicopathological characteristics in osteosarcoma

Clinicopathologic data	Case number	FN			αvβ3		
		High (*n* = 19)	Low/Neg (*n* = 41)	*P* value	High (*n* = 16)	Low/Neg (*n* = 44)	*P* value
Sex							
Male	38	11	27	NS	9	29	NS
Female	22	8	14		7	15	
Age (years)							
< 18	26	6	20	NS	8	18	NS
≥ 18	34	13	21		8	26	
Tumor size (cm)							
< 5	23	6	17	NS	4	19	NS
≥ 5	37	13	24		12	25	
Tumor location							
Tibia or femur	40	11	25	NS	11	29	NS
Other location	20	4	16		5	15	
Histologic subtype							
Conventional	55	17	38	NS	14	41	NS
Special	5	2	3		2	3	
Enneking staging							
I-IIA	21	4	17	NS	2	19	0.028
IIB	39	15	24		14	25	
Response to chemotherapy^a^							
Good	28	3	25	0.001	2	26	0.002
Poor	32	16	16		14	19	

*Neg* negative, *NS* no significance

^a^Good: tumor necrosis ≥ 90%, poor: tumor necrosis < 90%

**Table 4:** Association between co-expression of FN and αvβ3 and clinicopathological characteristics in osteosarcoma

Clinicopathologic data	Case number	FN^+^/αvβ3^+^ (*n* = 10)	Others^a^ (*n* = 39)	FN^−^/αvβ3^−^ (*n* = 11)	*P* value
Sex					
Male	38	7	20	11	0.011
Female	22	3	19	0	
Age (years)					
< 18	26	5	15	6	NS
≥ 18	34	5	24	5	
Tumor size (cm)					
< 5	23	3	16	4	NS
≥ 5	37	7	23	7	
Tumor location					
Tibia or femur	40	7	25	8	NS
Other location	20	3	14	3	
Histologic subtype					
Conventional	55	9	36	10	NS
Special	5	1	3	1	
Enneking staging					
I-IIA	21	2	11	8	0.013
IIB	39	8	28	3	
Response to chemotherapy*					
Good	28	0	20	8	0.002
Poor	32	10	19	3	

*NS* no significance

*Good: tumor necrosis ≥ 90%; poor: tumor necrosis < 90%; ^+^high expression; ^−^low/negative expression; ^a^FN^+^/αvβ3^−^ plus FN^−^/αvβ3^+^

### Association between expression of FN and αvβ3 in osteosarcoma and clinical outcome

The mean patient follow-up time was 45.2 months (range 8 to 86 months). By the end of the follow-up period, 28 (46.6%) patients survived with no evidence of disease, 13 (21.7%) remained alive with disease, and 19 (31.7%) succumbed to osteosarcoma (Additional file 1: Table S1).

**Fig. 3:**
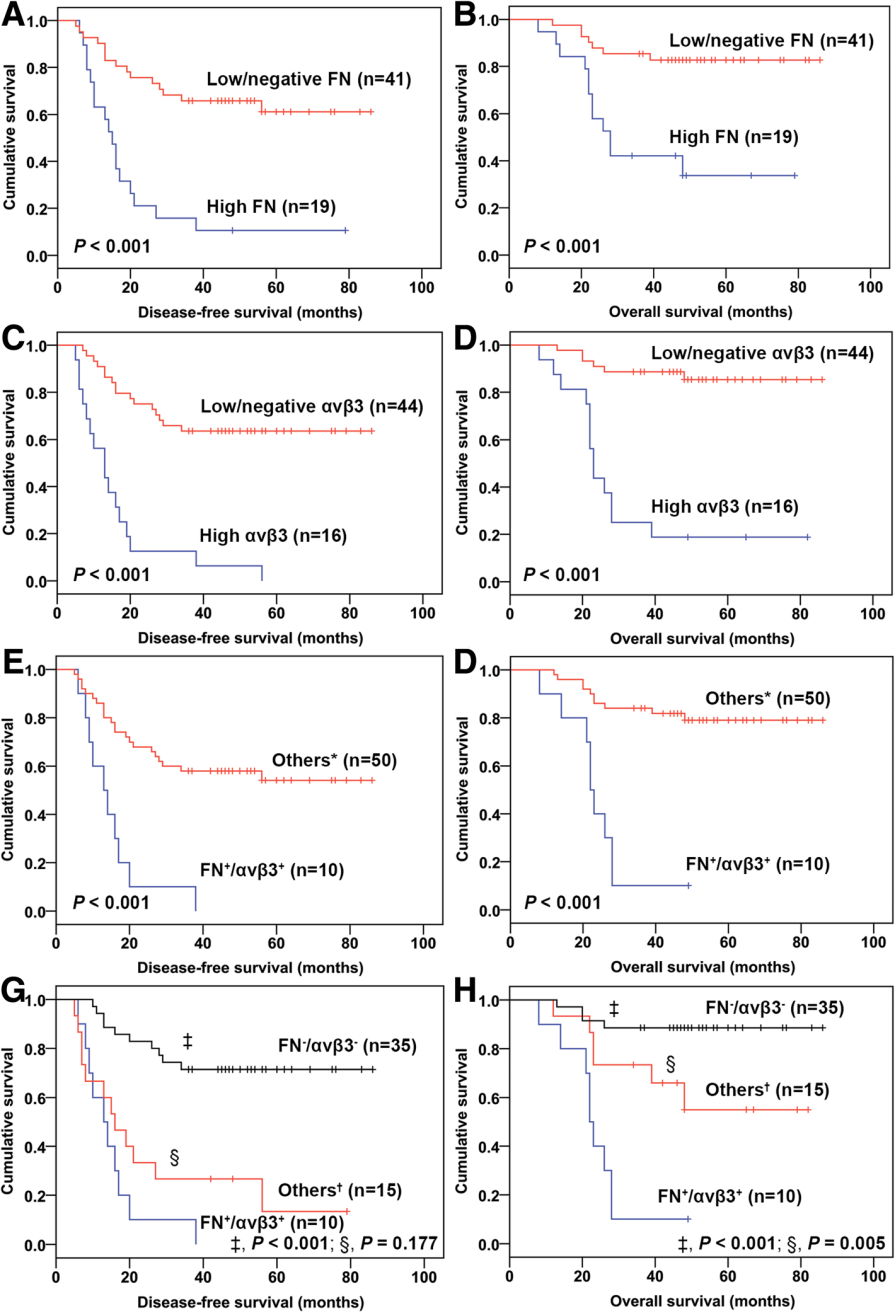
Kaplan-Meier analyses of disease-free survival (DFS) and overall survival (OS) time by FN and αvβ3 expression. **a**, **b** Significant differences in DFS (*P* < 0.001) and OS (*P* < 0.001) time were observed between high FN expression (FN^+^) and low/negative FN expression (FN^−^) groups. **c**, **d** Significant differences in DFS (*P* < 0.001) and OS (*P* < 0.001) time were observed between high αvβ3 expression (αvβ3^+^) and low/negative αvβ3 expression (αvβ3^−^) groups. **e**, **f** Significant differences in DFS (*P* < 0.001) and OS (*P* < 0.001) time were demonstrated between FN^+^/αvβ3^+^ and “others” groups. **g** A significant difference in DFS (*P* < 0.001) time was demonstrated between FN^+^/αvβ3^+^ and FN^−^/αvβ3^−^ groups. **h** Significant differences in OS time were demonstrated between FN^+^/αvβ3^+^ and FN^−^/αvβ3^−^ groups (*P* < 0.001) and between FN^+^/αvβ3^+^ and “others” groups (*P* = 0.005). *, “others” included the single high-expression and double low/negative-expression groups (FN^+^/αvβ3^−^ plus FN^−^/αvβ3^+^ plus FN^−^/αvβ3^−^); †, “others” included the single high-expression groups (FN^+^/αvβ3^−^ plus FN^−^/αvβ3^+^)

Univariate analysis of patient survival in relation to FN and αvβ3 expression levels is presented in Table 5, and survival curves are shown in Fig. 3. Mean DFS and OS times decreased with increasing expression of either FN (DFS, *P* < 0.001, Fig. 3a; OS, *P* < 0.001, Fig. 3b) or αvβ3 (DFS, *P* < 0.001, Fig. 3c; OS, *P* < 0.001, Fig. 3d). High expression of both FN and αvβ3 (FN^+^/αvβ3^+^) was associated with a shorter DFS time (*P* < 0.001, Fig. 3e) and OS time (*P* < 0.001, Fig. 3f) compared with the other groups (FN^+^/αvβ3^−^ plus FN^−^/αvβ3^+^ plus FN^−^/αvβ3^−^). Furthermore, the mean survival time decreased based on the extent of FN^+^ or αvβ3^+^ expression, which was longest for the FN^−^/αvβ3^−^ group (DFS, 67.14 months; OS, 78.43 months) followed by the single high-expression groups (FN^+^/αvβ3^−^ plus FN^−^/αvβ3^+^; DFS, 27.60 months; OS, 58.57 months) and then the FN^+^/αvβ3^+^ group (DFS, 15.10 months; OS, 24.10 months). The DFS time difference between the FN^+^/αvβ3^+^ group and FN^−^/αvβ3^−^ group (*P* < 0.001, Fig. 3g) as well as between the single high-expression groups (FN^+^/αvβ3^−^ plus FN^−^/αvβ3^+^) and the FN^−^/αvβ3^−^ group (*P* < 0.001) was statistically significant. A significant difference in OS time was also noted between the FN^+^/αvβ3^+^ group and the FN^−^/αvβ3^−^ group (*P* < 0.001, Fig. 3h), the single high-expression groups (FN^+^/αvβ3^−^ plus FN^−^/αvβ3^+^) and the FN^−^/αvβ3^−^ group (*P* = 0.022), and between the FN^+^/αvβ3^+^ group and the single high-expression groups (*P* = 0.005, Fig. 3h).

**Table 5:** Univariate analysis of FN and αvβ3 expression and osteosarcoma patient survival based on the log-rank test

Characteristics	Case number	Disease-free survival (months)					Overall survival (months)				
		Mean	SD	95%CI	*P* value		Mean	SD	95%CI	*P* value	
FN											
High	19	21.74	4.83	12.28–31.19	< 0.001		42.65	6.43	30.04–55.26	< 0.001	
Low/negative	41	61.08	5.19	50.91–71.25			75.16	3.75	67.81–82.52		
αvβ3											
High	16	16.06	3.34	9.51–22.62	< 0.001		33.38	6.08	21.45–45.30	< 0.001	
Low/negative	44	61.41	4.96	51.69–71.13			77.30	3.31	70.80–83.79		
FN^+^/αvβ3^+^	10	15.10	2.90	9.42–20.78	< 0.001		24.10	3.22	17.80–30.41	< 0.001	
vs. others^a^	50	55.60	4.91	45.98–65.22			73.31	3.61	66.23–80.39		
FN^+^/αvβ3^+^	10	15.10	2.90	9.42–20.78	< 0.001^b^	0.177^c^	24.10	3.22	17.80–30.41	< 0.001^b^	0.005^c^
FN^+^/αvβ3^−^ plus FN^−^/αvβ3^+^	15	27.60	6.75	14.36–40.84	< 0.001^b^		58.57	7.41	44.06–73.09	0.022^b^	
FN^−^/αvβ3^−^	35	67.14	5.09	57.16–77.13			78.43	3.57	71.43–85.43		

*SD* standard deviation, *CI* confidence interval

^+^High expression; ^−^low/negative expression; ^a^FN^+^/αvβ3^−^ plus FN^−^/αvβ3^+^ plus FN^−^/αvβ3^−^; ^b^vs. FN^−^/αvβ3^−^; ^c^vs. FN^+^/αvβ3^−^ plus FN^−^/αvβ3^+^

**Fig. 4:**
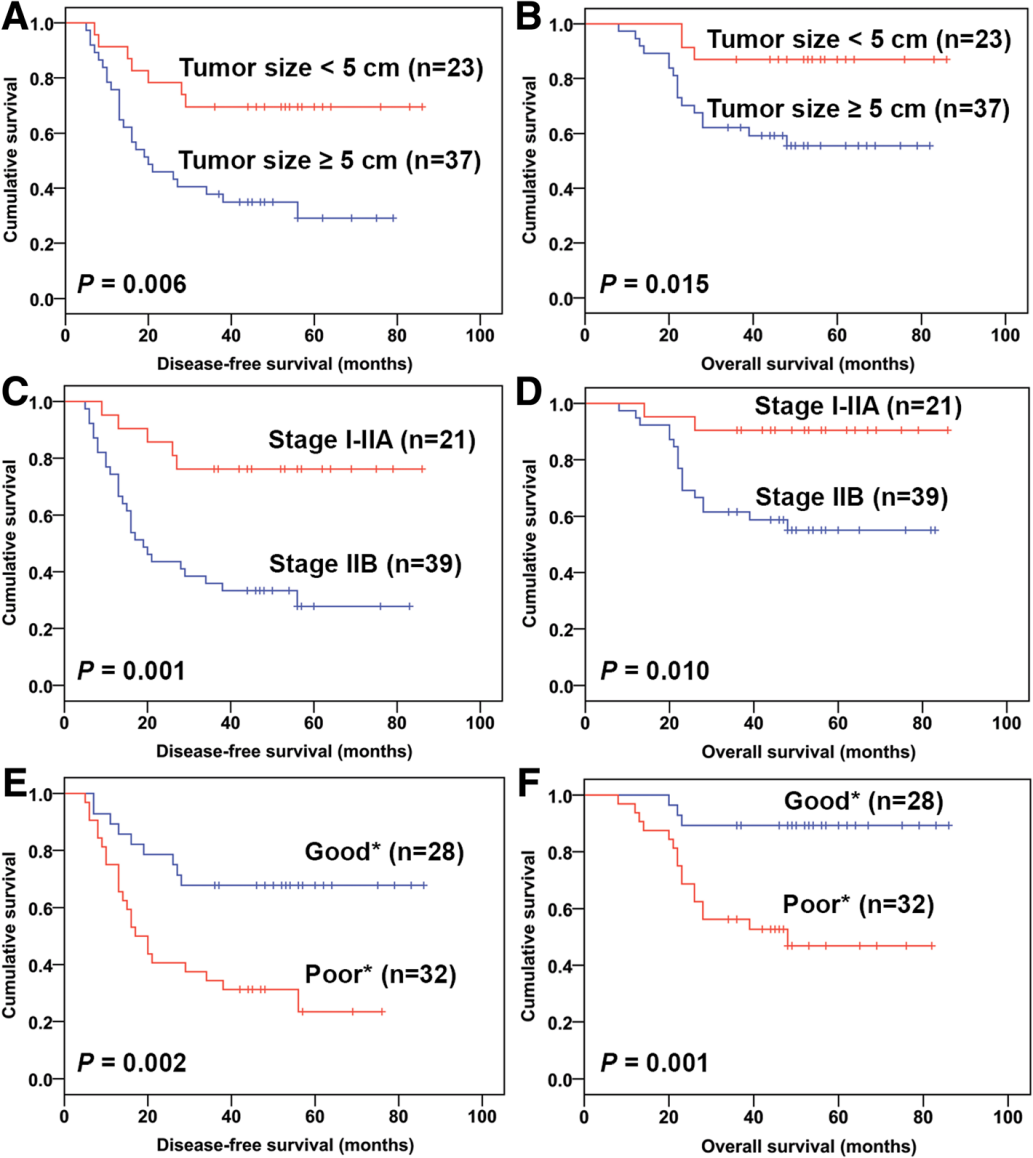
Kaplan-Meier analyses of disease-free survival (DFS) and overall survival (OS) time by clinicopathological features. **a**, **b** Significant differences in DFS (*P* = 0.006) and OS (*P* = 0.015) time were observed between large and small tumor sizes. **c**, **d** Significant differences in DFS (*P* = 0.001) and OS (*P* = 0.010) time were observed between Enneking stages I-IIA and IIB. **e**, **f** Significant differences in DFS (*P* = 0.002) and OS (*P* = 0.001) time were demonstrated between good and poor responses to chemotherapy. *Good: tumor necrosis ≥90%; poor: tumor necrosis < 90%

To explore the independent prognostic ability of FN and αvβ3 co-expression, three clinicopathological factors (i.e., tumor size, Enneking staging, and response to chemotherapy) that were significant predictors of survival time in univariate analysis (Table 6, Fig. 4) were evaluated by multivariate analysis. As shown in Table 7, according to multivariate analysis, FN^+^/αvβ3^+^ independently predicted worse DFS (hazard ratio (HR) = 2.66, *P* = 0.025, C-index = 0.75) and OS (HR = 3.75, *P* = 0.011, C-index = 0.84) of patients with osteosarcoma compared with other groups (FN^+^/αvβ3^−^ plus FN^−^/αvβ3^+^ plus FN^−^/αvβ3^−^). Compared with FN^−^/αvβ3^−^, FN^+^/αvβ3^+^ exhibited a statistically more significant predictive value for shorter DFS (HR = 5.62, *P* = 0.002, C-index = 0.79) and OS (HR = 6.35, *P* = 0.010, C-index = 0.85). Similarly, high expression of FN and αvβ3 individually (FN^+^/αvβ3^−^ plus FN^−^/αvβ3^+^) was significantly associated with worse DFS compared with FN^−^/αvβ3^−^. Therefore, co-expression of FN and αvβ3 was significantly correlated with poor DFS and OS in osteosarcoma patients.

**Table 6:** Univariate analysis of characteristics and osteosarcoma patient survival based on the log-rank test

Clinicopathologic data	Case number	Disease-free survival (months)				Overall survival (months)			
		Mean	SD	95%CI	*P* value	Mean	SD	95%CI	*P* value
Sex									
Male	38	53.81	5.59	42.85–64.76	NS	71.04	4.39	62.44–79.65	NS
Female	22	32.39	5.13	22.34–42.44		53.81	6.59	40.91–66.72	
Age (years)									
< 18	26	37.42	5.43	26.79–48.06	NS	51.51	4.42	42.84–60.17	NS
≥ 18	34	51.96	5.91	40.37–63.54		68.56	5.00	58.75–78.36	
Tumor location									
Tibia or femur	40	49.67	5.56	38.77–60.57	NS	67.47	4.52	58.60–76.33	NS
Other location	20	46.15	7.57	31.32–60.98		60.55	6.86	47.10–74.00	
Histologic subtype									
Conventional	55	49.20	4.77	39.86–58.54	NS	66.42	3.97	58.64–74.20	NS
Special	5	40.20	10.96	18.71–61.69		55.40	14.72	26.54–84.26	
Tumor size (cm)									
< 5	23	65.17	6.63	52.18–78.17	0.006	77.91	4.36	69.38–86.45	0.015
≥ 5	37	36.39	4.97	26.65–46.12		55.98	4.96	46.26–65.71	
Enneking staging									
I-IIA	21	70.05	6.27	57.75–82.34	0.001	79.71	4.25	71.39–88.04	0.010
IIB	39	36.42	5.08	26.46–46.38		56.54	4.87	47.00–66.07	
Response to chemotherapy^a^									
Good	28	63.86	6.14	51.83–75.89	0.002	79.11	3.76	71.74–86.48	0.001
Poor	32	32.84	4.91	23.22–42.47		51.56	5.42	40.94–62.18	

*SD* standard deviation, *CI* confidence interval, *NS* no significance

^a^Good: tumor necrosis ≥ 90%; poor: tumor necrosis < 90%

**Table 7:** Co-expression of FN and αvβ3 and osteosarcoma patient survival based on multivariate analysis

Characteristics	Comparison	Disease-free survival (months)			Overall survival (months)		
		HR	95%CI	*P* value	HR	95%CI	*P* value
FN^+^/αvβ3^+^	vs. others^a^	2.66	1.13–6.23	0.025	3.75	1.36–10.36	0.011
Tumor size (cm)	< 5 vs. ≥ 5	2.55	1.06–6.14	0.036	2.57	0.72–9.24	0.147
Enneking staging	I-IIA vs. IIB	3.34	1.20–9.28	0.021	2.90	0.60–13.87	0.184
Response to chemotherapy	Good vs. poor	0.75	0.30–1.84	0.526	0.48	0.12–1.99	0.309
C-index (95%CI)		0.75	0.65–0.86		0.84	0.70–0.97	
FN^+^/αvβ3^+^	vs. FN^−^/αvβ3^−^	5.62	1.91–16.52	0.002	6.35	1.54–26.14	0.010
FN^+^/αvβ3^−^ plus FN^−^/αvβ3^+^	vs. FN^−^/αvβ3^−^	3.79	1.50–9.54	0.005	2.31	0.59–8.96	0.228
Tumor size (cm)	< 5 vs. ≥ 5	2.04	0.84–4.93	0.115	2.39	0.67–8.56	0.182
Enneking staging	I-IIA vs. IIB	3.46	1.22–9.77	0.019	2.69	0.55–13.08	0.220
Response to chemotherapy	Good vs. poor	1.08	0.41–2.79	0.882	0.62	0.14–2.80	0.536
C-index (95%CI)		0.79	0.69–0.90		0.85	0.71–0.98	

*HR* hazard ratio, *CI* confidence interval

^+^High expression; ^−^low/negative expression; ^a^FN^+^/αvβ3^−^ plus FN^−^/αvβ3^+^ plus FN^−^/αvβ3^−^

## Discussion

Osteosarcoma is the second leading cause of cancer-related death in children and young adults due to its high metastatic potential [17]. Identification of tumor metastasis-associated biomarkers followed by development of promising therapies targeting molecular pathways will ultimately help to improve the prognosis of these patients. In this study, we found that high expression of FN or αvβ3 individually as well as their combined expression can serve as predictors for poor clinical survival among osteosarcoma patients.

The high level of FN expression in archived osteosarcoma tissues observed in our results was consistent with a previous study by Na et al. [18]. Additionally, osteosarcoma cells are better spread and have more actin stress fibers, when cultured with FN, compared with fetal bovine serum [19]. The correlation between FN and a poor response to chemotherapy of osteosarcoma found in the present study demonstrated that FN may support the aggressive potential of tumor cells. Overexpression of αvβ3 increases distant spread towards bone metastatic sites in various osteotropic tumors [20] and facilitates enhanced cell migration and metastatic potential in osteosarcoma [21]. In the present study, integrin αvβ3 was found to be upregulated in osteosarcoma and associated with advanced surgical stage and a worse response to chemotherapy, indicating the involvement of αvβ3 in treatment-resistant mechanisms.

Our results revealed that expression of FN or αvβ3 alone, as well as their co-expression, significantly contributes to the poor DFS and OS of osteosarcoma patients. Because it promotes tumor cell invasion and directional migration, FN acts as an independent unfavorable prognostic indicator for malignant tumors [4, 5, 22], and interaction between FN and αvβ3 may serve as a regulatory point to activate osteoblast adhesion and differentiation [13]. A previous study demonstrated that the FN-αvβ3 integrin axis promotes tumor cell migration, invasion, and metastasis by upregulating the activity of integrin-linked kinase [23], which is an independent prognostic factor for poor survival of osteosarcoma [24]. Thrombin-enhanced cell adhesion of osteosarcoma to FN can be inhibited by rhodostomin, which acts against integrin αvβ3 [11]. Therefore, multiple lines of evidence indicate that high FN and αvβ3 expression levels may contribute to the metastatic progression of osteosarcoma via various pathways. Antagonists targeting FN and αvβ3 are potentially able to increase the survival of patients with osteosarcoma. FN-targeted antibodies, such as L19-TNF [25] and F8-TNF [26], have demonstrated efficacy in inhibiting tumor growth and early pulmonary metastases of human osteosarcoma. Pending the results of ongoing studies, etaracizumab [27], a humanized version of LM609, which targets αvβ3, may represent another agent for the treatment of osteosarcoma.

Some limitations in the present study should be noted. First, selection bias should be considered due to the retrospective nature of the study. In addition, the application of neoadjuvant chemotherapy before surgery has a potential to influence the fidelity of FN and αvβ3 results. Our findings warrant further investigation into the quantitative analyses of FN and αvβ3 using biopsy specimens of osteosarcoma. Third, our study cohort included a relatively small number of patients, and the follow-up time for evaluating patient survival was relatively short. Thus, further large-scale studies with a longer follow-up time should be performed to offer more convincing evidence.

## Conclusions

Our study demonstrates that the expression of FN and αvβ3 is increased in osteosarcoma specimens and associated with poor clinical outcomes. Moreover, FN and αvβ3 co-expression is independently correlated with short DFS and OS. Hence, FN and αvβ3 may represent attractive therapeutic targets for the treatment of osteosarcoma. To improve the survival of osteosarcoma patients, further investigations are required to identify the prognostic significance in a larger population.

## Additional file

Additional file 1: **Table S1.** Clinicopathological characteristics of osteosarcoma. (DOCX 13 kb)

## Abbreviations

DFS: Disease-free survival
FN: Fibronectin
OS: Overall survival
