# Supplementary material for: Clinicopathological and prognostic values of fibronectin and integrin αvβ3 expression in primary osteosarcoma
Source: World J Surg Oncol. 2019 Jan 28;17:23. doi: 10.1186/s12957-019-1566-z (PMC6350278; doi:10.1186/s12957-019-1566-z)
Supplement: Supplementary file 1 — Table S1. Clinicopathological characteristics of osteosarcoma. (DOCX 13 kb) [file 12957_2019_1566_MOESM1_ESM.docx]

| **Table S1 Clinicopathological characteristics of osteosarcoma** | | |
| --- | --- | --- |
| Clinicopathologic data | n (%) |  |
| Sex |  |  |
| Male | 38 (63.3) |  |
| Female | 22 (36.7) |  |
| Age (years), mean (range) | 24 (4-55) |  |
| < 18 | 26 (43.3) |  |
| ≥ 18 | 34 (56.7) |  |
| Tumor size (cm) |  |  |
| < 5 | 23 (38.3) |  |
| ≥ 5 | 37 (61.7) |  |
| Tumor location |  |  |
| Distal femur | 22 (36.7) |  |
| Shaft of femur | 9 (15.0) |  |
| Humerus | 7 (11.6) |  |
| Tibia | 6 (10.0) |  |
| Pelvis | 4 (6.7) |  |
| Radius | 4 (6.7) |  |
| Proximal femur | 3 (5.0) |  |
| Scapula | 3 (5.0) |  |
| Maxilla | 2 (3.3) |  |
| Histologic subtype |  |  |
| Osteoblastic | 41 (68.4) |  |
| Chondroblastic | 8 (13.3) |  |
| Fibroblastic | 6 (10.0) |  |
| Small cell type | 3 (5.0) |  |
| Telangiectatic | 2 (3.3) |  |
| Enneking staging |  |  |
| I-IIA | 21 (35.0) |  |
| IIB | 39 (65.0) |  |
| Response to chemotherapy* |  |  |
| Good | 28 (46.7) |  |
| Poor | 32 (53.3) |  |
| Follow-up duration (month), mean (range) | 45.2 (8-86) |  |
| Survival outcome |  |  |
| Disease-free | 28 (46.6) |  |
| Alive with disease | 13 (21.7) |  |
| Succumbed to disease | 19 (31.7) |  |
| *Good: tumor necrosis ≥ 90%; poor: tumor necrosis < 90% | |  |
